# Proteomics Analysis to Explore the Resistance Genes of Silkworm to *Bombyx mori* Nuclear Polyhedrosis Virus

**DOI:** 10.3390/genes15010059

**Published:** 2023-12-30

**Authors:** Gui Ouyang, Heying Qian, Juan Sun, Runhuan Yang, Tao Gui, Wenbing Wang, Qiang Liu, Anli Chen

**Affiliations:** 1 College of Biotechnology, Jiangsu University of Science and Technology, Zhenjiang 212100, China; 2 Sericultural Research Institute, Chinese Academy of Agricultural Sciences, Zhenjiang 212018, China; 3 School of Medicine, Jiangsu University, Zhenjiang 212000, China; 4 Key Sericultural Laboratory of Shaanxi, Ankang University, Ankang 725000, China

**Keywords:** *B. mori*, BmNPV, resistance gene, 2-DE, qRT-PCR

## Abstract

The resistance of silkworms to *Bombyx mori* nuclear polyhedrosis virus (BmNPV) is controlled by a major dominant gene and multiple modifying genes. Given the presence of modified genes, it is difficult to determine the main gene by positional cloning. In this study, the main anti-BmNPV gene of BmNPV-resistant silkworm variety N was introduced into the susceptible variety Su to breed the near-isogenic line SuN with BmNPV resistance. The infection process of BmNPV in the hemolymph of Su and SuN was analyzed using the cell analysis system TissueFAXS PLUS. According to the law of infection and proliferation, hemolymph was extracted every 6 h for two-dimensional electrophoresis (2-DE) analysis and quantitative real-time polymerase chain reaction (qRT-PCR). Seven DEPs were found in comparisons between Su and SuN by 2-DE analysis. Among them, acid phosphatase, storage protein, and phenoloxidase can prevent pathogen invasion, which may play a role against BmNPV. Polyamine oxidase plays an important role in energy metabolism, which may be indirectly involved in the process of resisting BmNPV. Most of the transcriptional expression profiles of the seven DEPs were consistent with the 2-DE results. This study can provide a reference for the identification of anti-BmNPV genes and the breeding of BmNPV-resistant silkworm varieties.

## 1. Introduction

The domesticated silkworm, *B. mori*, is an important economic insect and lepidopteran model insect with a short breeding cycle and simple feeding mode [1,2]. As a traditional advantageous industry in China, the sericulture industry is an important economic source for thousands of sericultural farmers. However, silkworms are harmed by many kinds of silkworm diseases, such as nuclear polyhedrosis, cytoplasmic polyhedrosis, microsporidiosis, and bacterial septicaemia. Among these diseases, nuclear polyhedrosis caused by *B. mori* nuclear polyhedrosis virus (BmNPV) has the most serious impact on sericulture production. The loss caused by nuclear polyhedrosis accounted for more than 70% of the total loss of silkworm disease [3]. BmNPV has strong infectivity [4], high mortality [5], and limited chemical control effect. Therefore, finding anti-BmNPV genes, clarifying the molecular mechanism of resistance, and breeding silkworm strains with high resistance are urgently needed in sericulture production.

Recently, Huakang series [6,7,8] bred by the Sericulture Research Institute, Chinese Academy of Agriculture Science, Yunkang No. 1 [9,10] bred by the Sericultural and Apicultural Research Institute, Yunnan Academy of Agricultural Sciences, and Guican N2 [11] bred by Guangxi Sericulture Science Research Institute have a remarkable effect on resistance to BmNPV. Among them, Huakang and Yunkang 1 were bred by hybridization, backcrossing, selfing, and other breeding methods, using the highly BmNPV-resistant silkworm variety N with high tolerance to BmNPV as the female parent. The LC_50_ of the improved varieties was more than 10,000 times higher than that of the original varieties, and the resistance to BmNPV was significantly enhanced [6,7,8,9].

The breeding of these BmNPV-resistant varieties shows that the resistance of silkworm to BmNPV is controlled by a major dominant gene on the autosome and other modifying genes [12,13]. Lv [14] used single-nucleotide polymorphism markers for linkage analysis, which were obtained by genome sequencing of the highly BmNPV-resistant variety NB and the susceptible variety 306, and then found that the anti-BmNPV gene was located on chromosome 23 of silkworm. Gao [15] twice conducted linkage analysis with the backcross population of the highly BmNPV-resistant variety 99R and the more susceptible variety Dazao-N. However, the first linkage analysis result showed that the anti-BmNPV gene of 99R was linked to the chromosome 22, whereas the second did not. This finding indicated that linkage analyses of the anti-BmNPV gene were poor in repeatability. Wu [16] used the BmNPV-resistant silkworm variety AN for linkage analysis and mapped the anti-BmNPV gene on the chromosome 27. Then, Shao [17] located the anti-BmNPV gene in the 45 kb region of the chromosome 27, but failed to find genes related to BmNPV resistance among the 10 candidate genes. The abovementioned studies indicate that locating the main anti-BmNPV gene by forward genetics is difficult because of the presence of modified genes.

In addition to linkage mapping, using reverse genetics methods, such as transcriptome and proteome analysis, the researchers also screened and identified a large number of genes that may be involved in the resistance mechanism. Zhao [18] used two-dimensional electrophoresis (2-DE) to analyze the differentially expressed proteins (DEPs) between BmNPV-resistant and susceptible varieties of BmNPV infection and identified 36 proteins, of which 4 may be related to resistance. Bao [19,20] constructed cDNA libraries of the midgut, fat body, and hemolymph of the highly BmNPV-resistant variety KN and the susceptible variety 306 by suppressing subtractive hybridization, and obtained 158 differentially expressed genes. Li [21] obtained several differentially expressed genes in the fat body of the BmNPV-resistant variety Qiufeng N and the susceptible variety Qiufeng by transcriptome analysis, which were related to the cell membrane, metabolism, binding, and cell process. Qian [22] conducted metabolomic and transcriptomic studies on the midgut of the BmNPV-resistant variety Baiyu N and the susceptible variety Baiyu after infection with BmNPV. The results showed that the main difference between the two varieties was the metabolic pathway. In brief, the genetic regulation mechanism of silkworm’s resistance or sensitivity to BmNPV is complex, and the exact report on the main anti-BmNPV gene and its linked chromosome is still lacking.

In this study, the BmNPV-resistant silkworm variety SuN and its corresponding susceptible practical variety Su were used to detect the infection and proliferation of BmNPV virus particles in the hemolymph. Based on the result, hemolymph samples at appropriate time were selected to compare the protein expression differences between Su and SuN to obtain DEPs. The transcriptional expression profile of DEPs was constructed to verify the results of comparative proteomics analysis. The purpose of this study was to identify the DEPs between the hemolymphs of Su and SuN after BmNPV inoculation, and to excavate potential anti-BmNPV genes.

## 2. Materials and Methods

### 2.1. Silkworm Varieties and Virus Strains

The BmNPV-resistant silkworm variety SuN and the susceptible variety Su were provided by the Sericultural and Apicultural Research Institute, Yunnan Academy of Agricultural Sciences. BmNPV (Zhenjiang strain) was preserved by the Sericulture Institute, Jiangsu University of Science and Technology. SuN was obtained by crossing the resistant variety N with the susceptible variety Su, then backcrossing four times with Su as the male parent of the recurrent parent, and finally selfing and purifying. During each backcross, the new 2nd instar silkworms after ecdysis were fed with 10^8^ polyhedral inclusion bodies per ml (PIBs/mL) BmNPV to screen resistant individuals. Finally, most chromosomes of SuN were derived from Su, with only a few from N [7]. Compared with Su, the tolerance of SuN to BmNPV was significantly improved. Therefore, SuN is relatively pure and has stable resistance to BmNPV, which is an ideal genetic analysis material.

### 2.2. Virus Inoculation and Sampling

Fresh mulberry leaves with a flat surface were selected, and circular leaves with a diameter of 1 cm were made by using a puncher. A total of 4 μL of 10^8^ PIBs/mL BmNPV suspension was quantitatively added to each circular leaf. Then, 500 healthy 3rd instar silkworms of Su and SuN with similar body size were selected, and each silkworm was fed with one circular leaf after the surface of the leaves was air dried. The completely fed silkworms were selected for normal feeding and subsequent experiments.

Experimental sampling: After BmNPV inoculation, the hemolymph (60 μL of three silkworms) of Su and SuN was collected every 6 h from 48 h to 96 h for the detection of BmNPV proliferation (samples could not be taken after 96 h because although most individuals of SuN survived, all individuals of Su died and exhibited an outflow of white pus after 96 h). The midgut tissues (three silkworms) of Su and SuN were collected every 24 h, and the hemolymph (100 μL of three silkworms) of Su and SuN was collected every 6 h from 48 h to 96 h for 2-DE analysis. The hemolymph (500 μL of fifteen silkworms per sample, three biological replicates) of Su and SuN was collected every 6 h from 48 h to 90 h for qRT-PCR. An appropriate amount of antioxidant phenylthiourea was added to the hemolymph during sampling, and all samples were stored at −80 °C after quick freezing in liquid nitrogen.

### 2.3. Detection of BmNPV Proliferation in the Hemolymph

The slides and coverslips were washed with ultrapure water and wiped clean with dust-free paper. Each 10 μL of BmNPV diluent (10^8^ PIBs/mL) and hemolymph collected in Section 2.2 were taken to the slides with the coverslips flattened. The prepared samples were placed on the cell quantitative analysis instrument TissueFAXS PLUS (TissueGnostics, Vienna, Austria), and the TissueFAXS 6.0 system was used to manually establish the most suitable focal plane under the panoramic view in the bright-field shooting mode. The panoramic image of the hexagonal transparent hollow ring shape of virus particles was obtained, and nine 9.0000 mm^2^ (area size calculated by TissueFAXS 6.0) analysis areas were selected for each slide. After enhancing the discrimination between the image and the background, the hollow virus particles were identified and quantified by using StrataQuest 6.0. Considering that the actual viral load of the samples was correlated with the quantitative results, the standard curve was made by BmNPV diluents with different viral loads, and the actual viral load of the samples was calculated by the standard curve. The average and standard error of BmNPV particles in the nine analysis areas of each sample were calculated, and the broken line was drawn with Origin2022.

### 2.4. Protein Extraction and 2-DE

The tissue collected from Section 2.2 was quickly ground to powder with liquid nitrogen to obtain fully broken tissue cells. Each sample was added to 600 μL of hydration solution (48% urea, 2% CHAPS, 1.5% DeStreak™ Rehydration Solution (GE Healthcare, Chicago, IL, USA)), placed on ice for 1 h, and mixed every 10 min to cause complete lysis. Then, the sample was centrifuged at 12,000 rpm × 10 min, and the supernatant was placed on ice for 1 h and centrifuged at 12,000 rpm × 10 min again. The supernatants were protein samples and were used for 2-DE analysis after measuring the concentration by using the Bradford method.

Isoelectric focusing of 2-DE was performed on the BIO-RAD PROTEAN i12 IEF Cell (Bio-Rad, Hercules, CA, USA), with a 24 cm dry IPG strip (pH 3–10) and sample volume of 150 μg. The isoelectric focusing program was as follows: 60 V for 14 h, 200 V for 1 h, 500 V for 1 h, 2000 V for 1 h, and 8000 V for 6 h. After isoelectric focusing, the strip was placed in SDS equilibration buffer 1 (0.05 mol/L pH 8.8 Tris-HCl, 36% urea, 30% glycerol, 2% SDS, 0.002% bromophenol blue, 1% DTT) for 15 min and then in SDS equilibration buffer 2 (0.05 mol/L pH 8.8 Tris-HCl, 36% urea, 30% glycerol, 2% SDS, 0.002% bromophenol blue, 2.5% iodoacetamide) for 15 min. After reaching equilibrium, sodium dodecyl sulphate-polyacrylamide gel electrophoresis (SDS-PAGE) was performed using 12% separation gel. After SDS-PAGE, gel staining was performed by silver staining. Protein spots of DEPs were excavated, and the mass spectrometry identification was entrusted to the Testing Center of Beijing Protein Innovation Co., Ltd. The genes corresponding to the DEPs identified by mass spectrometry were searched in the silkworm database (http://sgid.popgenetics.net/index (accessed on 7 November 2022)).

### 2.5. RNA Extraction and qRT-PCR

The total RNA of hemolymph samples in Section 2.2 was extracted by using RNAiso Plus (Takara, Tokyo, Japan). After being detected by agarose gel electrophoresis, the qualified RNA was reverse transcribed into cDNA using the reverse transcription kit PrimeScript^TM^ RT reagent Kit with gDNA Eraser (Takara, Tokyo, Japan), which was then diluted five-fold for qRT-PCR.

Based on the CDS sequence of genes corresponding to the DEPs identified by mass spectrometry, specific primers were synthesized (Table S1). qRT-PCR was performed on the StepOnePlus realtime PCR system. The reaction system was as follows: 10 μL of FS Universal SYBR Green MasterRox (Roche, Basel, Switzerland), 8.2 μL of ddH_2_O, 0.4 μL of Primer F/R (10 μM), and 1 μL of cDNA template. qRT-PCR was performed as follows: 95 °C for 10 min, 40 cycles at 95 °C for 15 s, and 58 °C for 30 s, and then the melting curve was plotted at 65 °C with a gradient of 0.3 °C every 5 s to 95 °C. The silkworm housekeeping gene *Actin 3* (GenBank ID: NM_001126254) was used as the internal reference gene, and the obtained data were subjected to relative quantitative analysis using the 2^−ΔΔ*Ct*^ method [23]. Student’s *t* test was used to compare gene expression levels using SPSS19.0. The level of significance was set as follows: ns *p* > 0.05, * *p* < 0.05, ** *p* < 0.01. The expression profiles were drawn with Origin2022.

## 3. Results

### 3.1. Midgut Protein of Su Was Degraded Greatly after BmNPV Inoculation

**Figure 1:**
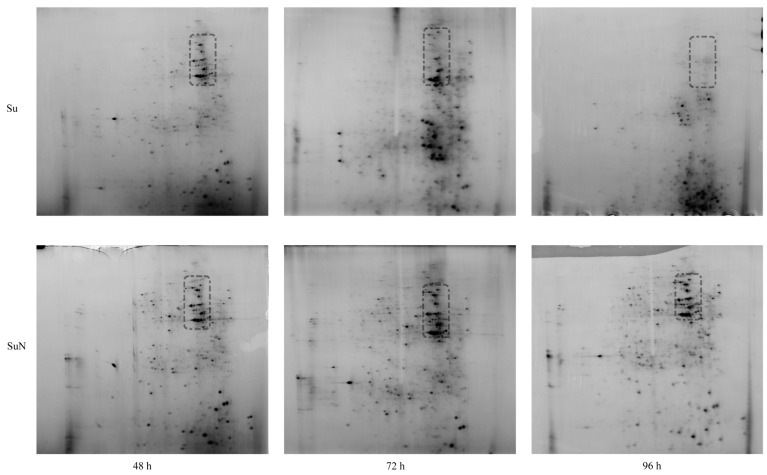
The expression changes of midgut proteins in Su and SuN after *B. mori* nuclear polyhedrosis virus (BmNPV) infection. The 2-DE maps of midgut proteins of Su and SuN at 48 h, 72 h, and 96 h after BmNPV inoculation are shown. The black dashed line enclose the macromolecular midgut proteins that were degraded in Su. The horizontal position of protein spots on the 2-DE map can reflect the isoelectric point of the protein, and the vertical position can reflect the molecular size of the protein. The higher the vertical position, the larger the molecular size of the protein.

The midgut tissue of silkworm was first infected after oral feeding of BmNPV, so the midgut proteins of Su and SuN were extracted for 2-DE analysis. The results showed that the midgut protein of Su was gradually degraded from 48 h to 96 h after inoculation with BmNPV. At 72 h, the number and the expression abundance of macromolecular midgut proteins in Su were greatly reduced, with lots of middle-molecular proteins appearing. At 96 h, the macromolecular midgut proteins in Su were almost completely degraded, the middle-molecular proteins were reduced, but a large number of small-molecular proteins appeared. By contrast, the midgut protein of SuN had not been degraded during the whole process (Figure 1).

It took only 48 h for the midgut proteins of Su to be degraded from appearance to basically complete degradation, indicating that the damage of BmNPV to silkworm midgut was severe and rapid. After BmNPV infection, the midgut proteins of silkworm rapidly degraded and produced a large number of small-molecule degraded proteins, which directly affected the identification of target DEPs. Therefore, obtaining real DEPs by using the midgut protein for 2-DE analysis after BmNPV infection is difficult.

### 3.2. BmNPV Proliferated Rapidly in the Hemolymph of Su after Infection

**Figure 2:**
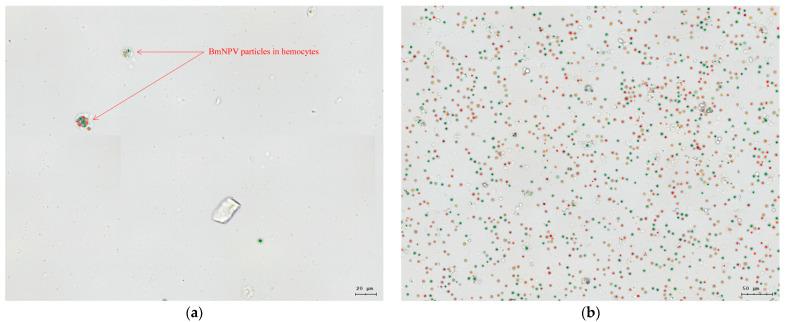
BmNPV particles entered the hemocytes of Su and proliferated. (**a**) BmNPV entered the hemocytes of Su at 78 h after inoculation. (**b**) The hemocytes of Su were lysed completely and the BmNPV particles proliferated rapidly at 96 h after inoculation. The colored round-like particles in the figures are virus particles detected by the TissueFAXS PLUS, and the irregularly shaped translucent substances are hemocytes or cell debris.

**Figure 3:**
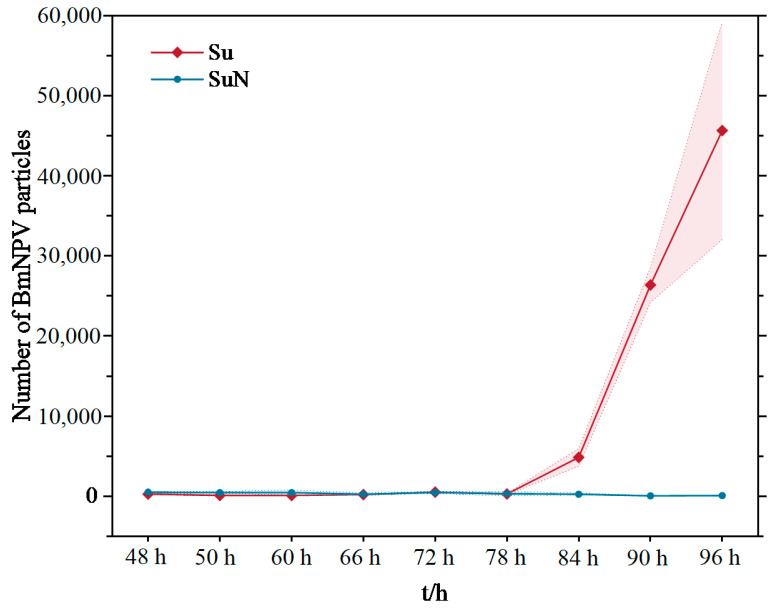
Proliferation of BmNPV in the hemolymph of Su and SuN. The broken line shows the number of BmNPV particles in Su and SuN’s hemolymph at different time points. Each polyline data point was the average of nine analysis areas, and the range of dashed lines around the broken line indicated the standard deviation. The actual number of virus particles was more than that scanned by the TissueFAXS PLUS because the hemolymph cannot be completely compressed into monolayer cells during hemolymph sample preparation.

The midgut and hemolymph of silkworms are important tissues for BmNPV infection. Compared with the midgut, the component of hemolymph is relatively simple. Therefore, the TissueFAXS PLUS cell analysis system was used to analyze the infection and proliferation of BmNPV in the hemolymph of Su and SuN. The results showed that the virus particles were detected in the hemocytes of Su at 78 h after BmNPV inoculation (Figure 2a). The hemocytes of Su were lysed, and the virus particles were released into the hemolymph at 84 h. Virus particles proliferated rapidly in the hemolymph of Su over time, and then a large number of virus particles were scattered in the hemolymph at 96 h (Figure 2b). Contrary to Su, virus particles were never detected in the hemolymph of SuN after BmNPV inoculation (Figure 3).

The proliferation of BmNPV in the hemolymph showed that the virus particles occurred in a short time from infecting hemocytes to rapid proliferation in the hemolymph. If the samples were taken every 24 h for 2-DE analysis, then multiple important time points would be missed. Therefore, the hemolymph of Su and SuN was collected every 6 h for 2-DE analysis after BmNPV inoculation.

### 3.3. Expression of Hemolymph Proteins in Su and SuN after BmNPV Inoculation

**Figure 4:**
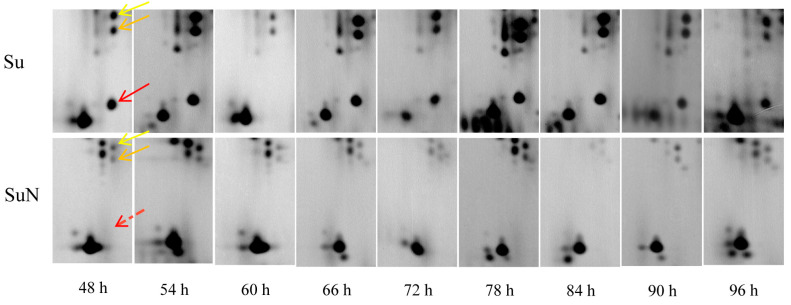
The expression changes of S1, S2, and S3 in hemolymph of Su and SuN after BmNPV infection. S1 is indicated by red arrow, S2 is indicated by yellow arrow, and S3 is indicated by orange arrow.

**Figure 5:**
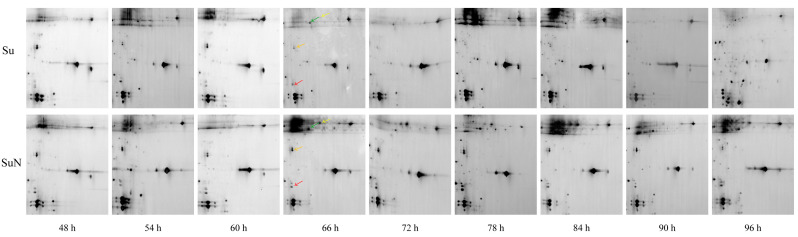
The expression changes of SN1, SN2, SN3, and SN4 in hemolymph of Su and SuN after BmNPV infection. SN1 is indicated by red arrow, SN2 is indicated by orange arrow, SN3 is indicated by yellow arrow, and SN4 is indicated by green arrow.

**Figure 6:**
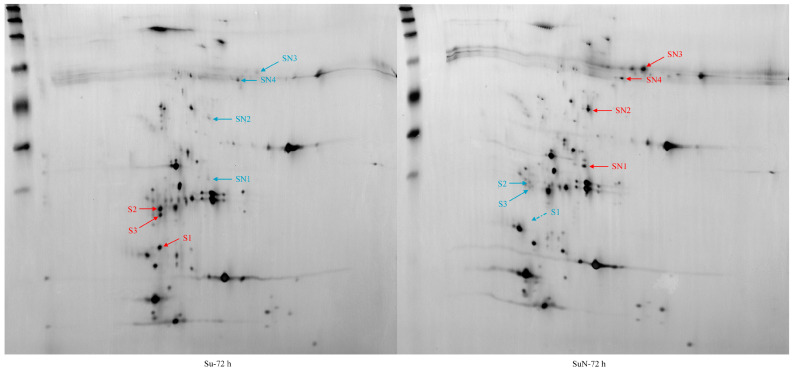
All DEPs in hemolymph of Su and SuN at 72 h for example. The red arrow indicates that the expression abundance is relatively higher, and the blue arrow indicates that the expression abundance is relatively lower.

The 2-DE results of hemolymph protein showed that one DEP (namely, S1) was detected in the hemolymph of Su at all time points except for 60 h, but this protein was not detected in the hemolymph of SuN (Figure 4). Except for S1, no significant difference in hemolymph protein was observed between Su and SuN at 48 h, 54 h, and 60 h after BmNPV inoculation. At 66 h, 72 h, 78 h, 84 h, and 90 h after BmNPV inoculation, two DEPs (namely, S2 and S3) were found in the hemolymph of Su, and their expression in Su was remarkably higher than that in SuN (Figure 4). Moreover, the other four DEPs (namely, SN1, SN2, SN3, and SN4) were found in the hemolymph of SuN, and their expression in SuN was markedly higher than that in Su (Figure 5 and Figure 6).

The information of DEPs was obtained through searching in the silkworm database (Table 1). The DEP S1 is the hemolymph juvenile hormone binding protein (hJHBP) of silkworms, and the corresponding gene number is *KWMTBOMO13811*. S2 is uncharacterized protein LOC105842978, and the corresponding gene number is *KWMTBOMO03739*. S3 is uncharacterized protein LOC101740628, and the corresponding gene number is *KWMTBOMO03745*. SN1 is an acid phosphatase, and the corresponding gene number is *KWMTBOMO08187*. SN2 is a polyamine oxidase, and the corresponding gene number is *KWMTBOMO06255*. SN3 is a storage protein, and the corresponding gene number is *KWMTBOMO13992*. SN4 is the pro-phenol oxidase, and the corresponding gene number is *KWMTBOMO09392*.

### 3.4. Transcriptional Level Analysis of Genes Corresponding to DEPs

**Figure 7:**
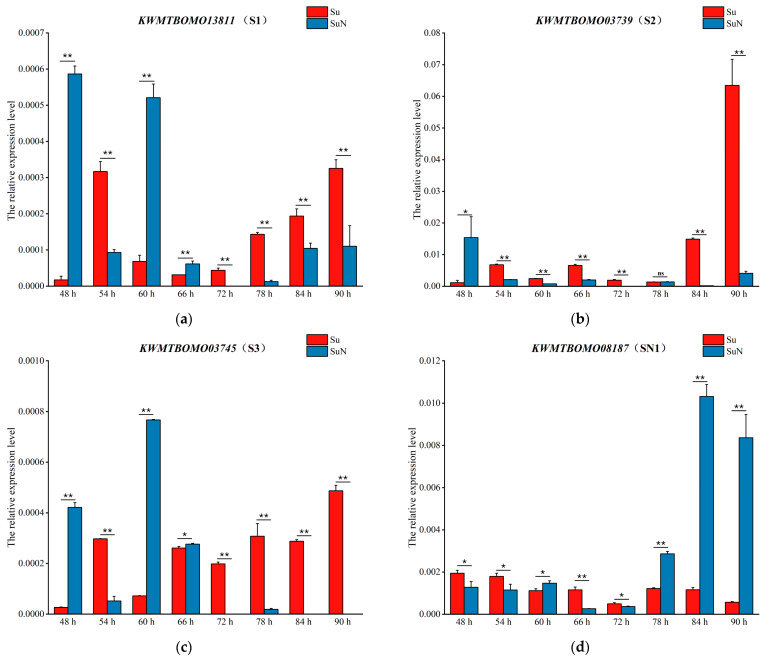

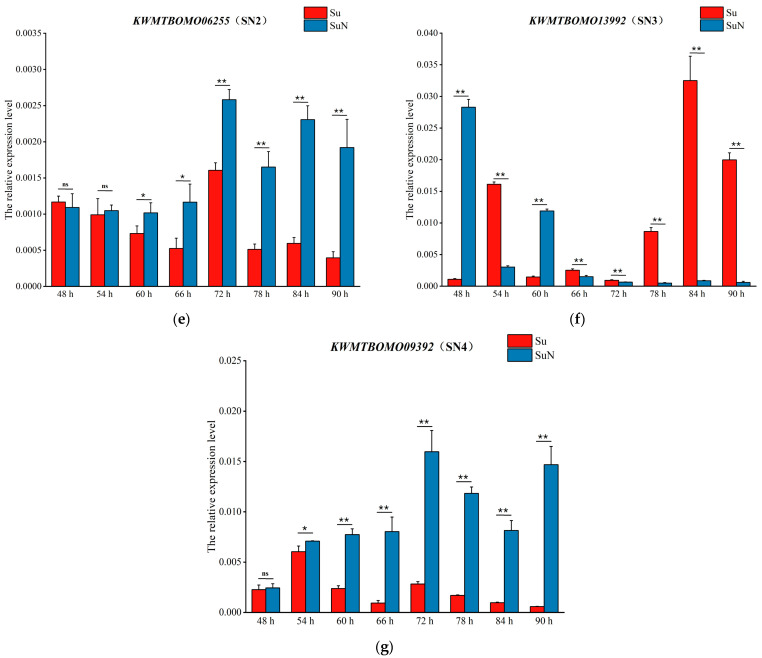
Expression profiles of genes corresponding to the seven DEPs. (**a**) *KWMTBOMO13811*, S1; (**b**) *KWMTBOMO03739*, S2; (**c**) *KWMTBOMO03745*, S3; (**d**) *KWMTBOMO08187*, SN1; (**e**) *KWMTBOMO06255*, SN2; (**f**) *KWMTBOMO13992*, SN3; (**g**) *KWMTBOMO09392*, SN4. Error bars indicate the standard error of the mean (*n* = 3). Significant differences were assessed using Student’s *t*-test (ns *p* > 0.05, * *p* < 0.05, ** *p* < 0.01).

In verifying the results of 2-DE, qRT-PCR was performed to detect the transcription of the genes corresponding to DEPs in the hemolymph of Su and SuN at different time points after BmNPV inoculation. The results showed that the transcription level of *KWMTBOMO13811* in SuN was remarkably higher than that in SuN at 48 h, 60 h, and 66 h after BmNPV inoculation, and the transcription level in SuN was considerably higher than that in SuN in the other five time points (Figure 7a). The transcription level of *KWMTBOMO13992* in SuN was markedly higher than that in SuN at 48 h and 60 h after BmNPV inoculation, and the transcription level in SuN was remarkably higher than that in SuN at the other six time points (Figure 7f). *KWMTBOMO13811* and *KWMTBOMO13992* were located on chromosome 23. The expression trend of these two genes was consistent at all time points except for 66 h, but the qRT-PCR results were inconsistent with the 2-DE results. However, it can be determined that chromosome 23 of SuN was derived from N, which can provide a reference for the subsequent determination of the chromosome where the main anti-BmNPV gene is located. The qRT-PCR results of *KWMTBOMO03739* (Figure 7b), *KWMTBOMO03745* (Figure 7c), *KWMTBOMO08187* (Figure 7d), *KWMTBOMO06255* (Figure 7e), and *KWMTBOMO09392* (Figure 7g) were consistent with the 2-DE results.

## 4. Discussion

The midgut of silkworms is the main digestive organ and an immune barrier against microbial invasion and proliferation [24,25]. After oral feeding, BmNPV initially infects the midgut, then spreads to the tracheal cells through the midgut, and finally spreads to the hemolymph, resulting in a fatal infection [26,27]. When we sampled, at 48 h after inoculation with BmNPV, the midguts of Su were basically normal. At 72 h, the midguts of Su were loose, fragile, and slightly damaged. At 96 h, the midguts of Su were severely ulcerated, which seemed to be a symptom of protein degradation. Differential gene expression generally only upregulates expression or downregulates expression to cause differences, and there is rarely a phenomenon that gene expression is completely silenced [28,29]. In our study, many macromolecular midgut proteins of Su disappeared on the 2-DE map at 96 h. Thus, we speculate that viral infection leads to the gradual degradation of midgut proteins, rather than the inhibition of macromolecular protein expression and the promotion of small molecule protein expression. During 2-DE analysis, a large number of macromolecular proteins were degraded in the midgut of Su after BmNPV infection, resulting in many non-real DEPs. However, the hemolymph protein did not degrade during the entire infection cycle, so it was more suitable for DEP analysis. Within a short period of 18 h, the virus particles occurred from infecting hemocytes to rapid proliferation. Therefore, the previous method of sampling every 24 h was not suitable for the study; extracting hemolymph every 6 h for 2-DE analysis is more reasonable.

Silkworms are metamorphosis insects, which must undergo 3–4 molts from larvae to pupate. During larval molting, juvenile hormone (JH) and ecdysone play a key regulatory role. hJHBP is the carrier of JH, which plays an indispensable role [30]. At about 60 h of the 3rd instar, silkworms began to enter the feeding reduction period to prepare for molting. At this time, the JH decreased, and the MH began to increase, which explained why hJHBP was not detected in the hemolymph of Su at 60 h after BmNPV inoculation. In our study, hJHBP was not detected in the hemolymph of SuN, but SuN larvae could develop normally. In addition, the qRT-PCR results showed that hJHBP was normally transcribed in SuN. Therefore, *KWMTBOMO13811* of SuN might undergo variable splicing, resulting in a change in the molecular weight or isoelectric point of the hJHBP. Furthermore, the hJHBP could not be detected at the same position, but its ability to bind to JH was not affected.

After breaking through the midgut barrier and infecting silkworm cells, BmNPV virus particles often cause systemic immune response, resulting in a large number of proteins binding to each other to serve the invasion or antagonism mechanism between the virus and the host [31]. The protein functions of *KWMTBOMO03739* and *KWMTBOMO03745* remain unclear, but based on GO analysis (https://www.ebi.ac.uk/QuickGO, (accessed on 24 June 2023)), both of them participate in membrane structure and protein binding. After BmNPV inoculation, the number of BmNPV particles increased exponentially in the hemolymph of Su, but hardly increased in the hemolymph of SuN. It can be inferred that the proteins encoded by *KWMTBOMO03739* and *KWMTBOMO03745* respond to the invasion and proliferation process of BmNPV. Thus, the transcription level and protein expression of *KWMTBOMO03739* and *KWMTBOMO03745* in Su were significantly higher than those in SuN.

Acid phosphatase is involved in thiamine metabolism, riboflavin metabolism, and metabolic pathways. Studies have shown that acid phosphatase in bee venom has anti-inflammatory, antibiosis, and antinociceptive immune promotion and other functions [32]. Polyamine oxidase is a FAD-dependent enzyme, which is an integral part of the polyamine interconversion cycle and plays an important role in energy metabolism [33,34]. Storage proteins are insect hemolymph-specific proteins, which are primarily synthesized in fat bodies and released into the hemolymph, and such proteins play an important role in insect metamorphosis and egg development. Some storage proteins are responsive to pathogenic infection, and they can even suppress pathogen multiplication [35]. Phenoloxidases are ubiquitous in all living organisms and are closely related to various activities such as the biosynthesis of pigments and neurotransmitters (dopamine), defense responses, and tissue hardening [36]. Acid phosphatase, polyamine oxidase, storage protein, and phenoloxidase were abundantly expressed in the BmNPV-resistant variety SuN, indicating that they may be related to the anti-BmNPV mechanism. Acid phosphatase, storage protein, and phenoloxidase can prevent pathogen invasion, which may have a certain effect on resistance to BmNPV infection, and polyamine oxidase plays an important role in energy metabolism, which may be indirectly involved in the process resistant to BmNPV. However, whether they are the main resistance genes to BmNPV remains to further study.

The BmNPV-resistant variety SuN is a near-isogenic line of Su with BmNPV resistance, which was bred by introducing the resistance gene of the anti-BmNPV variety N into the susceptible silkworm variety Su. After four rounds of backcrossing with male Su as described in Section 2.1, around 96.875% (1−(1/2) ^n+1^, n = 4) of the chromosome of SuN was theoretically derived from Su, and the remaining part was derived from N [7]. The resistance of SuN is derived from this small part of N. According to the genetic material relationship between Su and SuN, identifying DEPs and determining the chromosomes which the corresponding genes located on through 2-DE analysis and mass spectrometry identification, in theory, the chromosome which the main anti-BmNPV gene located on can be inferred. In this study, the genes corresponding to the DEPs were located on five chromosomes (Chr7, Chr11, Chr14, Chr16, Chr23), indicating that the main anti-BmNPV gene of SuN may be located on these five chromosomes. Lv [14] found that the resistance of the highly BmNPV-resistant variety NB to BmNPV showed a dominant single-gene control and mapped the main anti-BmNPV gene on chromosome 23. Based on this finding, Nie [37] also mapped the resistance gene to a region containing 22 candidate genes on chromosome 23 of silkworm. In this study, *KWMTBOMO13811* and *KWMTBOMO13992* are located on chromosome 23, which confirms the results of Lv and Nie. This study can provide a reference for the positional cloning of the main anti-BmNPV gene of silkworms.

## 5. Conclusions

In this study, the BmNPV-resistant variety SuN, which is the near-isogenic line of the susceptible variety Su, was bred. The rapid degradation of midgut protein and the exponential proliferation of virus particles in the hemolymph were observed in Su after BmNPV infection. Seven DEPs were identified between the hemolymph of Su and SuN by 2-DE analysis and mass spectrum. Among them, acid phosphatase, storage protein, and phenoloxidase can prevent pathogen invasion, which may play a role against BmNPV. Polyamine oxidase plays an important role in energy metabolism, which may be indirectly involved in the process of resisting BmNPV. This study provides a reference for the identification of anti-BmNPV genes and the breeding of BmNPV-resistant silkworm varieties.

## Figures and Tables

**Table 1:** Gene annotation of DEPs in Silkworm Genome Database.

DEPs	Genes	Chromosome Position	Gene Annotation in Silkworm Genome Database
S1	*KWMTBOMO13811*	Chr23: 10000380–10005184 (+)	hemolymph juvenile hormone binding protein precursor [*Bombyx mori*]
S2	*KWMTBOMO03739*	Chr7: 371497–372518 (−)	Uncharacterized protein [*Bombyx mori*]
S3	*KWMTBOMO03745*	Chr7: 422709–423596 (+)	Uncharacterized protein [*Bombyx mori*]
SN1	*KWMTBOMO08187*	Chr14: 1161961–1192896 (+)	Venom acid phosphatase Acph-1-like [*Papilio xuthus*]
SN2	*KWMTBOMO06255*	Chr11: 3382416–3398048 (+)	Probable polyamine oxidase 5 [*Bombyx mori*]
SN3	*KWMTBOMO13992*	Chr23: 15551943–15556861 (−)	sex-specific storage-protein 1 precursor [*Bombyx mori*]
SN4	*KWMTBOMO09392*	Chr16: 360567–373088 (+)	Pro-phenol oxidase [*Bombyx mandarina*]

## Data Availability

Data are contained within the article and Supplementary Materials.

## Supplementary Materials

The following supporting information can be downloaded at: https://www.mdpi.com/article/10.3390/genes15010059/s1, Table S1: Primers used in qRT-PCR.
