# Supplementary material for: Proteomics Analysis to Explore the Resistance Genes of Silkworm to Bombyx mori Nuclear Polyhedrosis Virus
Source: Genes (Basel). 2023 Dec 30;15(1):59. doi: 10.3390/genes15010059 (PMC10815149; doi:10.3390/genes15010059)
Supplement: Supplementary file 1 [file genes-15-00059-s001.zip › genes-2753918-supplementary.pdf]

Table S1. Primers used in qRT-PCR.

Primer name	Forward Primer sequence (5′-3′)	Reverse Primer sequence (5′-3′)
Actin 3	GGATGTCCACGTCGCACTT	GCGCGGCTACTCGTTCACT
KWMTBOMO13811（S1）	TGATGATAACGGAGTTCAAC	TGATATAGCACGCTTCAGCT
KWMTBOMO03739（S2）	CATCGGAGCAAACGATCAC	GTCTTAAGAGCACGAACTGT
KWMTBOMO03745（S3）	CGAAGCTGAGCACGTTGAAA	TCTCTGTTTGGTTCCTGAAC
KWMTBOMO08187（SN1）	GTTCAGCAATGGGATGAGTC	CAACTGCATGACGTCCAGGT
KWMTBOMO06255（SN2）	GCACGTCTATAGGAGTTGT	GTTCCGGTATAGTGAAGTGT
KWMTBOMO13992（SN3）	CTGGTCAACGGAAAGAACAC	GATGACGTACATCTGCATCT
KWMTBOMO09392（SN4）	AACACGCTGAACACGCTGT	TACTCTTACGTCGGATTGTGT
